# Serious gaming for graduates employability enhancement

**DOI:** 10.3389/fpsyg.2023.1324397

**Published:** 2023-12-15

**Authors:** Nacim Yanes, Ikram Bououd, Leila Jamel, Nazik Alturki

**Affiliations:** ^1^College of Computer and Information Sciences, Jouf University, Sakaka, Saudi Arabia; ^2^RIADI Laboratory, La Manouba University, Manouba, Tunisia; ^3^Kedge Business School, Marseille, France; ^4^Department of Information Systems, College of Computer and Information Sciences, Princess Nourah bint Abdulrahman University, Riyadh, Saudi Arabia

**Keywords:** soft skills, serious games, graduates, employability, higher education, quantitative, qualitative

## Abstract

Higher Education Institutions (HEIs) are blamed for being insouciant about the business world and not efficiently steeling students with employment abilities. As a reply to this exigency from business owners, HEIs have started to show commitment to graduate employability through developing and adopting new educational material, such Serious Games (SGs), to help students acquire these soft skills. This paper explores the students’ perceptions of the significance of SGs on soft skills and therefore boosting graduate employability. We carried out a quantitative and qualitative study with 322 students from business colleges in the aim to focus on the impact of user experience in SGs on critical thinking and teamwork. The results highlight the strong influence of SGs to acquire these soft skills.

## 1 Introduction

Nowadays, the curriculum graduates’ outcomes and the students’ soft skills in today’s labor market are one of the most discussed debates in higher education management (Clarke, 2017; Chiara and Wieandt, 2019; Al Asefer and Abidin, 2021). Soft skills or people skills are defined as interpersonal qualities or personal attributes that become important to consider in job applications (Robles, 2012; Cimatti, 2016; Marin-Zapata et al., 2022). Archer and Davison (2008) reported that the International Employee Barometer (IEB) survey confirmed the significance of employers ‘soft skills. They pointed out that employers give higher importance to soft skills compared to higher institutions graduates’ qualifications. Currently, business leaders emphasize soft skills development and acquisition in job application just like hard skills. They consider that soft skills have a critical influence on productive performance in workplaces (Nealy, 2005; Robles, 2012).

Likewise, the World Economic Forum distinguished 10 out of 16 key skills in the twenty-first century to be related to employees’ soft competencies, mainly teamwork, critical thinking, innovation, etc. (Deloitte Access Economics, 2017). Business competes within globalization, quick development of technologies and demographic movies, which gives an importance to soft skills. Indeed, the Deloitte Access Economics (2017) report predicts that compared to jobs in 2000, soft skill intensive occupations will balance from one half to two-thirds of all jobs by 2030. Companies feel the relevance of developing soft skills of their employees to harness broader benefits. In the same report, it is indicated that businesses spend $4 billion on training, and an additional $7 billion each year on hiring the right staff.

In the education domain, employers and decision makers have criticized HEIs for not being involved enough to prepare students properly for the current workforce, and thus are continuously emphasizing students’ deficiency of soft skills. Even though HEIs seem to respond to this criticism and address this issue, enhancement in students’ acquisition of soft skills is still to be missing and needs more investigations (Chiara and Wieandt, 2019; Warrner, 2021).

A new way to promote the students’ soft skills consists of developing and adopting new educational material, namely Serious Games (SGs). In fact, there has been a rising focus on how SGs can be used to support significant objectives of the academic environment, like teaching, training, collaborating, and learning (Sanchez et al., 2009; Sanchez and Olivares, 2011; Corrigan et al., 2014; Devitt et al., 2015; Hamari et al., 2015; Iten and Petko, 2016; Dimitriadou et al., 2021; Krath et al., 2021). Several research confirmed that SGs may be an important teaching tool, as they are interactive, engaging, and immersive activities (Shaffer and Gee, 2006; Devitt et al., 2015; Almeida and Simoes, 2019; Assaf et al., 2019; Calabor et al., 2019). The incorporation of SGs as an innovative learning material in HEIs-mainly in business schools-aims at enhancing students’ engagement and performance (Lohmann et al., 2019). Recent research findings are highlighting several advantages of SGs such as promoting active learning, motivation (Connolly et al., 2012; Chan and Zary, 2019; Garneli et al., 2021; Gaurav et al., 2022), increasing creativity, social interactions (Goh and Wasko, 2009; Anastasiadis et al., 2018; Flood et al., 2018; Zhonggen, 2019; Krath et al., 2021) and even soft skills such as leadership, team building, collaboration, and critical thinking (Sanchez et al., 2009; Sanchez and Olivares, 2011; Corrigan et al., 2014; Devitt et al., 2015), (Almeida and Simoes, 2019).

The expansion of SGs usage with the growing skills and expectations of the new generation of learners are the main motivators toward the incorporation of such games in the educational process (Almeida and Simoes, 2019; Assaf et al., 2019). Despite the optimism that these games are promoting soft skills (Dondlinger, 2007), high quality studies supporting these conveys are qualitative rather than quantitative (Mayer et al., 2004; Steinkuehler and Duncan, 2008; Connolly et al., 2012). According to Connolly et al. (2012) and Solinska-Nowak et al. (2018) quantitative studies about the impact of SGs on developing high order soft skills are needed.

Although the impact of SGs on hard skills and know-how requested by the workforce has received increased attention over the last decades, their impact on soft skills is still under-explored by research studies in higher education. This paper contributes to the existing literature by answering the following research questions:

- To which extent does user experience in SGs foster students’ development of soft skills including critical thinking and teamwork?
- To which extent this experience may impact their individual and team performance?

The current paper presents an empirical study run with a business game with students from business colleges in France and Saudi Arabia Universities. 322 candidates participated in the study, 154 from governmental universities in KSA and 168 from Kedge Business School, in France. We analyzed and discussed the results obtained. Then students were asked to fill in a quantitative questionnaire and respond to a set of open-ended questions in the aim to have an overview about their opinions about the game experience. Our findings bring better insights about whether SGs are fostering soft skills.

This paper is organized into five sections. The background section is dedicated to a literature review about soft skills development and graduate’s employability. Then we introduced the research model linking different constructs. The methodology is presented in section three. Section four is dedicated to quantitative analysis and the presentation of the results. Finally, we discussed our findings and their implications, and we concluded with insights, limitations, and directions for future research.

## 2 Literature review

### 2.1 Outcome-based education and importance of soft skills

During the last century, an important criticism has evolved to HEIs to be unable to equip students with the adequate hard skills and soft skills. It has been asserted that to accomplish a positional benefit, students must develop basic/technical skills and transferable/soft skills (Clarke, 2017). In response, Outcome-based Education (OBE) was proposed as a theory that built each part of an educational system around outcomes. An outcome is defined as the abilities that a student can obtain at the end of a learning experience, and which should be observable and measurable (Rao, 2020). Teaching is then designed to engage students in learning activities so the achieving rate of those outcomes may be increased (Kandlbinder, 2014; Jadhav et al., 2020). Faculties are expected to act as facilitators for students in a comfortable learning and teaching environment to develop the competencies that the curriculum expects to boost (Senaratne and Gunarathne, 2019). Ciappei and Cinque (2014), mentioned in that soft skills are hard to define since they can have different forms according to different contexts, and they keep developing throughout the entire lifetime. Several designations are used to characterize soft skills including life skills (WHO, 1993), transferable skills (Yorke, 2006), twenty-first century skills (Moore and Morton, 2017), generic competences, transversal skills besides key competencies for an effective life and an active society (Chiara and Wieandt, 2019). In the literature, soft skills are classified according to the level of standing within the company (i.e., junior, manager and executive) or to the action scope (i.e., personal, or social; Chiara and Wieandt, 2019).

In this work we adopt the definition given in Haselberger et al. (2012) which qualifies soft skills as “a dynamic combination of cognitive and meta-cognitive skills, interpersonal, intellectual and practical skills and ethical values, Authors stated that soft skills may guide people to act positively so that they may handle efficiently with the defiance of their business life. Examples of soft skills are team working, problem solving, communication abilities, critical thinking, creativity, self-confidence, etc.

In the OBE learning context soft skills are course outcomes enabling students to choose, adapt, and apply other skills to a variety of situations and across several social situations (Haselberger et al., 2012; Gaurav et al., 2022). As we mentioned above there is no unique style of teaching or assessment is an OBE learning process. Faculties have the entire freedom and responsibility in writing course outcomes that students must achieve, designing suitable assessments and instruction (i.e., how instructors may help students to get the ability to do what they are expected to; Rao, 2020). A new way to promote the students’ soft skills consists in developing and adopting new educational material, namely Serious Games. SGs are taking an expanding place in education as a new approach to teach complex concepts and skills to students. Thanks to their design mixing seriousness and fun, research reported an enhancement in students’ motivation and perceived performance compared to traditional learning materials.

Many HEIs adopt SGs as teaching strategies to attract new generations of students who increasingly use diverse and sophisticated tools. SGs not only permit a mastering of hard skills, but they have the potential to make students develop several soft skills. Caeiro-Rodríguez et al. (2020) reported that HEIs need to shape students to be critical thinkers, innovators, problem solvers, effective collaborators within a variety of social contexts and groups, and self-learning to remain at the forefront of their fields.

For instance, in MMOG (Massively Multiplayer Online Games), guilds are formal groups allowing players to concretize their strategic collaboration. Social and soft skills are being learnt and practiced when playing games. Several pro-social behaviors have been witnessed in these games like positive social skills, generosity, helpfulness, creativity and task motivated play and self-regulation (Lenhart et al., 2008), empathy (Narvaez et al., 2008), leadership and teamwork (Yee, 2006; Goh and Wasko, 2009). Goh and Wasko (2009) argued that MMOGs are complex social entities containing player-founded organizations and reflecting real-life. The authors also investigated attempts to identify potential leaders based on social network analysis. They found that guilds (player-founded organizations) need leaders with success key skills like mediating conflicts, planning, controlling, motivating and management. Authors reported that academic and business worlds are interested in such leaders. IBM and Google are investigating leadership features and their pertinence to management practice.

According to Yee (2006) in MMOG advancement often necessitates increasing the level of collaboration between players, which strengthens their team cohesion and communication skills. Lenhart et al. (2008), argued that SGs provided teenagers with significant opportunities for social learning and exchange where players help each other, make decisions that impact the game and the players’ groups, and discuss moral and communal issues. Games mastery and knowledge are increasingly becoming a considerable part of adolescents’ subculture. It forms a part of their social capital and influences the nature of their subgroups (Yee, 2006; Goh and Wasko, 2009). Peterson et al. (2008), showed that SGs help players get skills and abilities such as: designing strategic plans, solving problems, logical thinking, attention, focusing, high memorizing, creativity, team working, decision-making and reasoning.

SGs in education can stimulate creativity and productivity among learners (Flores, 2015). In this study, authors used many gaming tools such Brainspace, Duolingo, Socrative and Class-Dojoto to enhance second language (L2) learning process. The results showed that this helped L2 learners improve their speaking, writing, and reading comprehension skills while encouraging participation and teamwork. According to the NMC Horizon Report and as mentioned in Yanes and Ikram (2019) education professionals are beginning to embrace gamification because they understand how well-made games can greatly increase students’ inventiveness and efficiency. The authors concentrated on based-games tools competences for learning English. A SWOT analysis gaming tool was used through brainstorming sessions with students enrolled in Jouf University in KSA. The authors reported that educational video games foster critical thinking, involvement, problem solving, innovation and teamwork–all of which contribute to the development of remedies to issues associated with society and the environment. Corrigan et al., also paid attention to how games affected the development of cooperation in taking decisions in airports (Corrigan et al., 2014). In fact, they developed a game entitled “SKYBOARD” as a component of their Airport Collaborative Decision Making (A-CDM) system. In the game, four principal players, mainly the ground handler, the airline, the ATC, and the airport were up against several obstacles to face to free the aircraft. SKYBOARD was a creative product of the A-CDM systems as it was a highlight component that betters communication, collaboration and decision-making and productivity among all stakeholders. The game was integrated into many other learning and learning programs in the airport which aims the strengthening of partnership and communication between multi-agency contributors. Devitt et al. (2015), proposed an online resource; designed as a serious game; to real-world marketing principles for undergraduate students enrolled in a marketing course during the 2013–14 academic year. The study used a group-based simulation game (MMX) where students must work in groups to manage their own business while battling with other teams in a specific marketplace. So, to optimize the business’s key performance indicators, they must also develop a marketing strategy built on core marketing concepts. The game appeared to facilitate favorable communication and negotiation soft skills as well as development of critical thinking and cognitive knowledge application.

Sanchez and Olivares (2011) proposed a problem-solving and collaborative serious game for students which is centered about the evolution of species from science class. The experiments used Mobile based Serious Games (MSG) in Chili schools with students in the 8th level learning and comprehending scientific theories. An in-home MSG evolution game was designed where players (students) play with units of specific species and the system controls these species as enemies. MSGs “BuinZoo” and “Museum” (Sanchez and Olivares, 2011) were also used to enable the study of ideas linked to species evolution knowledge. The main idea of these MSGs is to fix a mission related to an issue that students must solve during the museum visit. The issue is in relation to the process or concepts of the evolution of species. The game is based on individual/ group multiple choices questions. At the beginning the player (student) gives an individual answer. When the answer is correct, then the game offers additional details regarding the solution and a hint for responding to the group queries. When it comes to group questions, the instructions tell students that they must meet as a group to discuss and select the right answer because every group member knows the answer to the question. After completing every question in the game, students receive a notification reminding them to create a presentation with multimedia outlining the lessons they acquired during their visit and resolving the initial issue. These MSGs included time, resources, and logic constraints as in a real commercial environment and participated in developing students’ collaboration and problem-solving skills.

Di Loreto et al. (2013) in their first research suggested a “Do not Panic” game that supports crisis training and to stop the largest panic attack that humanity has ever witnessed. The game’s idea is that is that every participant starts out as an integral part of a worry control group that has to work together to calm others down. The game uses (i) contents based on real facts and incidents pertaining to handling crisis and (ii) rules which encourage the player to apply the “best practices” associated with soft skills related. Then the authors developed a mobile game entitled “MoDo” which is designed to be played in groups in a real-world setting using smartphones and technologically enhanced objects. The game’s principle is that every team must finish its mission–evacuating all individuals within a building or zone before other teams and this using fixed time and limited resources. It follows that in a constrained amount of time, each team must “collect” and evacuate the greatest number of individuals. For this to happen, the team needs to return the “collected” individuals to the building’s or zone’s entrance. Such serious games promoted soft skills, basic procedures of learning and collaboration.

Cheng et al. (2016) studied how SGs features impact students’ learning process, with a focus on engagement and pleasure, and the measurement of perceived enhancement of soft skills. In this study, undergraduates taking courses related to networking management and data communications were asked to play a CISCO telecommunication-based SG. The game’s primary goal is to assist players in understanding and practicing the concept of binary numbers by using multiple formats for converting. With each right answer, one conversion is eliminated, and the total number of conversions rises. Since the game screen is filled with unfinished conversions, players are forced to finish every conversion fast. Even though they are involved in the game with identical difficulty scale, students may have difficulties with diverse scales. At the end of these experiences, students wrote reports and filled-in an online survey regarding their standpoints of participating in serious gaming. The study findings confirmed SGs present big advantages on undergraduates’ ability to think critically and analyze, take decisions, and solve problems, and furthermore on their attitudes, i.e., engagement and pleasure, during learning process.

Business owners are also interested in soft skills values and recognize what these attributes can offer to their enterprises. SGs have been shown to improve students’ hard skills and to raise learning efficiency, commitment, and determination (Caeiro-Rodríguez et al., 2020). Although, research dealing with the effect of SGs on learning experience, which in turn improves the students’ perceived soft skills, is sparse.

### 2.2 Graduates’ employability

Hurrell et al. consider graduate employability as a set of accomplishments, abilities, and personal characteristics that make graduates more likely to get employment and succeed in their chosen occupations (Hurrell et al., 2013). Several studies focused on finding graduate at-tributes and skills to boost (Mezhoudi et al., 2021; Saidani et al., 2022) and predict graduates’ employability. Horwitz et al. (2013) found that communication is one of the topmost ranked competencies in the employment market. While others observed that interpersonal characteristics or soft skills had the greatest influence on employment (Grugulis and Vincent, 2009; Nickson et al., 2012). Butum and Nicolescu (2019) considered that teamwork, communication, and respect (People skills or relational skills) are the most that contribute to effective performance and adaptability in responding to changing circumstances. In higher education, researchers noted that graduates who possess a set of employability skills, knowledge, and personal qualities are more likely to find employment and succeed in their chosen fields, which is advantageous for them as well as for the workforce, the community, and the economy (Cruz et al., 2020; Römgens et al., 2020). Graduates and their supervisors were given access to a 51-item questionnaire that was produced and contained a selection of employability abilities. The results obtained showed that the skills with the greatest mean weighted discrepancy scores were those related to problem solving, information technology, adjusting to change, and risk taking, in line with the supervisors’ perceptions. Graduates believed that problem-solving and quick decision-making were the abilities with the highest mean weighted disparity scores and the greatest need for curricular improvement. Saibon and Kamis (2019) stated that graduates in business management must be proficient in six employability skills: communication, information technology application, leadership and management, teamwork, interpersonal and entrepreneurial abilities in addition to analytical and problem-solving. In this study we focus on graduates’ employability, soft skills-critical thinking, team working, and individual performances required by the business industry.

In the following we describe the research model and the hypotheses. Then, we present the methodology and the results of this study. Then we debate the findings of this work and their repercussions for HEIs. Finally, we give the limitations of this work and future improvement suggestions.

## 3 Research model and hypothesis

### 3.1 User experience

**Figure 1:**
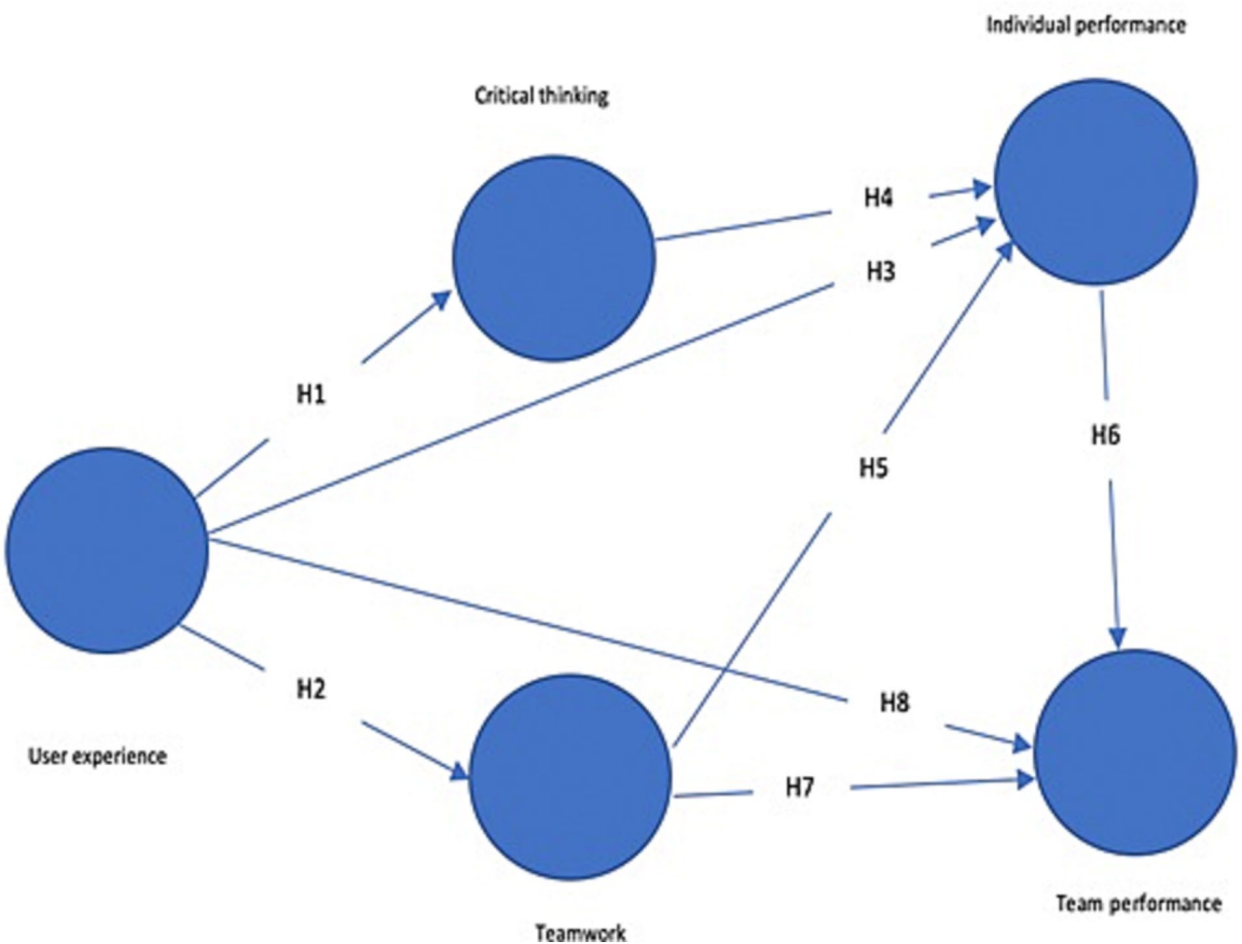
Research model.

The construct “User experience” has its roots in Human-Computer interfaces (Moizer et al., 2019). According to the international standard on Ergonomics of human system interaction, it’s defined as the user’s perceptions and responses resulting from the use or the anticipated use of a product, system, or a service (Dis, 2009). The research model adopted here, as represented in Figure 1; is based on this perception. In fact, Moizer et al. (2019) argued that user experience in serious games is a complex construct that contains five components: gaming experience, learning experience, adaptivity, usability and fidelity.

- Gaming experience: this dimension describes the relationship of the user with the game, the amount of flow, attachment, challenge, and the ability to perform tasks.
- Learning experience: this dimension is correlated to the game effectiveness to make the user learn via its learning goals.
- Adaptivity: this dimension reflects the capacity of the game to adjust itself with the needs, the goals, and the background of learners.
- Usability: this dimension is correlated to the ease of use, the control, and the adoption of the game.
- Fidelity: this dimension refers to the degree of realism that the game provides to the user.

### 3.2 Critical thinking

Critical thinking is defined as a deep assessment of a claim or situation to find a suitable answer and level of confidence in accepting, rejecting, or withholding judgment (Piel, 2008; Moore and Morton, 2017). According to Valenzuela et al. (2011) this construct contains two main components: expectancy and task value.

- Expectancy corresponds to the assumption that a person has about achieving a task appropriately.
- Task value contains four elements: cost, interest, attainment, and utility.

Banutu-Gomez (2004), considered critical thinking as a tremendous skill required by leaders and followers. The author argued that leaders should work on improving their followers’ critical thinking in the aim to foster creativity and succeed as a team. Additionally, various researchers confirmed that this feature is a relevant component for effective leadership (Ashkanasy, 2002; Banutu-Gomez, 2004; Rosenzweig, 2007; Piel, 2008; Valenzuela et al., 2011) that in its turn leads to enhanced satisfaction and commitment among team workers (Walumbwa et al., 2005). According to Ducheneaut et al. (2007), in WOW, higher players’ levels have much more social experience compared to other players with lower levels. Gentile et al. (2019) reported that serious games foster teamwork and collaborative learning, and could develop deep reasoning, critical thinking, complex systems, etc. We address the H1 hypothesis:

*H1*: User experience with serious games has a positive impact on critical thinking.

### 3.3 Teamworking

“Teamwork is the ability to work together toward a common vision. It is the fuel that allows people to attain uncommon results, Andrew Carnegie (1835–1919). According to Ducheneaut et al. (2007) team-working and many other soft/social competencies/skills could be learnt through serious games. They argued that MMOG teamwork is relevant to achieving hard levels (Goh and Wasko, 2009; De Freitas and Routledge, 2013). Players should increase their collaboration and interdependence especially when various characters could supplement each other’s advantages and weaknesses in the aim to make a stronger group and fight in a guild (Yee, 2006).

Several games endorse cooperation between players to attain the game goal. Cooperation is a manner to synergize efforts when all players are combatting the same enemy or when players want to share beneficial information and expertise (e.g., the power of re-search). Successful teamwork is the result of mutual knowledge sharing and strong commitment between team members. Fostering collaboration and teamwork generates a high satisfaction with expertise and an intense achievement sentiment (Carenys et al., 2017). The following hypothesis is treated:

*H2*: User experience with serious games has a positive impact on team-working.

### 3.4 Individual performance

The usage of technology as learning materials in serious games would allow individuals to assist themselves in the performance of tasks (Goodhue and Thompson, 1995). Serious games allow mixing entertainment with serious content in a crucial way so learners can experience learning-by-doing (Alvarez and Rampnoux, 2007). Goodhue and Thompson (1995) assert that experience with technology influences individual performance. In addition, technology is very crucial to support skills development and foster the effectiveness of teams (Griffith et al., 2003). Consequently, we can propose the following hypotheses:

*H3*: User experience in serious games has a positive impact on individual performance.

Soft skills encompass, including critical thinking and teamwork enable employees to work effectively with others, adapt to new challenges, and contribute positively to the organization’s culture (Haselberger et al., 2012; Gaurav et al., 2022). Furthermore, critical thinking is impacting individuals working in teams (Hollenbeck et al., 1995; Natale and Ricci, 2006). It is positively impacting individuals, teams, and organizations. In fact, critical thinking enhances communication inside the team and fosters individual learning effectiveness (Boyd and Fales, 1983). We make the following hypothesis:

*H4*: Critical thinking has a positive impact on individual performance.

*H5*: Teamwork has a positive impact on individual performance.

### 3.5 Team performance

Employees with strong soft skills are better equipped to handle challenges, communicate clearly, and build strong relationships with colleagues and clients (Haselberger et al., 2012). For instance, individuals are influenced by the context and the team in which they are working. Podsakoff et al. (1997) assert that altruistic and helping behavior in teamwork is positively impacting both quantity and quality aspects of individual and team performance. Further, team performance is considered as the sum of the individual performance of each team member and even more complicated in the context of mutually dependent team members (Sonnentag and Frese, 2001).

*H6*: Individual performance has a positive impact on team performance.

*H7*: Teamwork has a positive impact on team performance.

*H8*: User experience in serious games has a positive impact on team performance.

## 4 Methodology

In the aim to better understand the impact of user experience in serious games on soft skills acquisition and students’ satisfaction, we conducted a quantitative study. We used structural equation modeling using SmartPLS software. We supported this study with a qualitative analysis of an open-ended question with the aim of having an overview of students’ opinions about the gaming experience. The survey was created by using Google forms and the link was sent to students who were informed about the purpose of the study and that no personal information was being gathered. A brief introduction also notified the respondents about the survey’s contents and purpose. The students’ consent to complete the questionnaire for the purposes of this study was obtained. We conducted this study with 322 Bachelor students enrolled in business schools in Saudi Arabia and France. The business game is considered as an important encounter with precise learning goals focused upon teambuilding, initiation with this new learning environment. Consequently, students were invited to take a survey as a part of their post-class assessment. 322 students provided us with relevant and useful responses to be analyzed in this study.

The serious game is developed by a company called ARKHE (the same name as the game). The protocol started with a manual detailing rules and procedures for the general conduct of the game. Several rules were associated with advanced functions will be dis-covered as far as students’ take decisions and make their own business evolve. The main idea of the game is to produce different types of boats. They may order raw materials, buy machines, hire manpower, rise salaries, foster advertising, and manage their stock.

Students are dispatched in groups of 6 or 7 students. Mixed groups (multinational) are mandatory, and the organization was defined randomly by the professors before the beginning of the game. Students should discuss in English in groups and with their teachers. Each group represents a company who is trying to evolve with competitive companies with the same initial condition. Teachers accompany students as coaches, administrators, or consultants. They may play the role of bank employee who studies a loan demand so students must make an appointment and convince him to accept their request. The game was played over 4 days (30 h). It was programmed over 1 year of business activity. After every month the condition and the market state could change. The difficulty is rising and so the challenges do. After every decision, coaches announce the groups rankings and the results. Students were evaluated based on several criteria: manufactory, profitability, cash flow management, human resource management, customer satisfaction and stock management.

In the intent to study eventual game’s impact on different soft skill acquisition, we make students respond to the questionnaire twice. The first time after the decision in the middle of the game and the second was after the decision at the end of the game. We created two groups before/after in the aim to better understand the real impact of the game on the students.

## 5 Quantitative study

### 5.1 Research method and measures

To study the impact of gaming experience (user experience in serious games), critical thinking, teamwork, individual performance, and team performance, we conducted a quantitative study. The survey was created to investigate the hypotheses proposed by the model of the research. In Table 1, we present the constructs’ measurement and their corresponding items from the literature. The reference of each measurement is given. Finally, we tried to adapt the different scales to the serious gaming context. We use abbreviations as follows: User experience (UE), Critical Thinking (CT), Individual performance (IP), Team performance (TP) and Team Working (TW) Table 1.

**Table 1:** Constructs with correspondent items and measurement references.

Constructs and references		Construct items
User experience in Serious Games (Moizer et al., 2019)	Gaming experience	The gaming experience was challenging. I found the serious game stimulating. I was able to achieve the goals of the game. I remained focused on the game throughout. The gaming experience was immersive. The overall gaming experience was positive. Interactions in the game were fraught and tense.
	Learning’s experience	The learning goals of the game were clear. The game scenario had relevance to the issue of communication skills development. The serious game required me to use my communication skills. The serious game provided opportunities to receive feedback. I recognize the value of serious games as a tool for learning. Using online role play is an appropriate way to develop communication skills.
	Adaptivity	The professor interventions were helpful in focusing my attention
	Usability	The professor’s interventions helped to develop my confidence. The pace of the role play was too fast. The interface of the game was easy to use. It was easy to get started with the serious game. I learnt how to use the serious game quickly. The user manual was well written and clear The survey function aided my reflection. The gesture and mood functions were useful. It was easy to move around. It was easy to create dialog. It was easy to respond to in-game survey questions.
	Fidelity	The game environment was visually appealing. I can identify with the story/scenario in the serious game. The experience felt real.
Critical thinking (Valenzuela et al., 2011)	Expectancy	Concerning reasoning correctly, I am better than most of my classmates. I feel able to understand everything related to thinking in a rigorous way. I can learn how to think in a rigorous way. I can learn how to reason correctly better than most of my classmates.
	Task value	It is important for me to learn how to reason correctly. It is important for me to be good at reasoning. It is important for me to use my intellectual skills correctly. It is important for me to be good at solving problems. Critical thinking will help me to become a good professional. Critical thinking will be useful for my future. Critical thinking is useful in everyday life. Critical thinking is useful for other subjects and courses. I like to reason properly before deciding about something. I like to learn things that will improve my way of thinking. I like thinking critically. I like to reason in a rigorous manner. If I have a problem that requires me to reason in a critical way, I am disposed to sacrifice the time that I would otherwise have devoted to other things. I am disposed to sacrifice quite a lot of time and effort to improve my way of reasoning. It is worth investing time and efforts to acquire and use critical thinking.
Teamworking (Lower et al., 2015)		I feel confident in my ability to work in a team. I know how to give my team members feedback that will not hurt their feelings. I ask others for feedback. I make an effort to include other members of my group. I value the contributions of my team members. I treat my team members as equal members of the team. I am good at communicating with my team members. I feel confident in my ability to be a leader.
Individual performance (Choi et al., 2010)		My deliverables are of excellent quality. I manage time effectively. I meet important deadlines on time
Team performance (Choi et al., 2010)		The team’s deliverables are of excellent quality. The team managed time effectively The team met important deadlines on time

### 5.2 Data collection

Data was collected from Bachelor students who played the business game in two sessions December 2022 and June 2023. 322 answers were gathered and useful for the analysis step from Kedge Business School and Saudi universities. No ethical approval is requested for this work.43,7% answers are men (56,3% are women), aged between 19 and 21. 2,5% have already used business games more than 3 times, 1,7% have already used them 2 times, 40,3% have already used them for one time and 55,5% were playing for the first time.

The following analysis is reported on the complete sample (before and after the end of the game).

### 5.3 Data analysis and findings

As data was collected in cross-cutting contexts, data gathered may be exposed to bias. However, the Common Method Variance (CMV) percentage (24%) is acceptable and fitting to data collection conditions. We used the SmartPLS software for quantitative analysis. First, we tested construct validity and ultimately, we investigated the hypotheses (structural model).

#### 5.3.1 Measurement model

First, we started by testing convergent validity, by illustrating the link between each construct and its items. We tried to show to what extent every item captures the essence of the corresponding construct. To do so, we used the Cronbach (α) reliability measure and focused on items’ cross loadings. The results are provided in Table 2. Then, we tested discriminant validity which means that every item has a stronger link to its construct compared to any other construct in the model.

**Table 2:** Cronbach alpha and cross loadings.

	UE	TP	CT	TW	IP
Cronbach α	0,957	0,903	0,962	0,899	0,836

To check discriminant validity, we compared in Table 3 the square root of Average Variance Extracted (AVE) to the interrelationship between each construct with the rest of the model’s constructs. All the AVE values (in diagonals) are greater than 0.5 and every AVE is higher than any correlation with other constructs. In addition, all constructs have firmly established discriminant validity, as the Heterotrait-Monotrait Ratio is below 0.90 (Hair et al., 2022). Results show that convergent and discriminant validity are well-established to all constructs (Table 4).

**Table 3:** Discriminant validity (Fornell-Larcker criterion).

	CT	IP	TP	TW	UE
CT	**0.776**				
IP	0,474	**0.868**			
TP	0,446	0,739	**0.915**		
TW	0,664	0,491	0,505	**0.767**	
UE	0,603	0,427	0,458	0,602	**0,719**

The bold values are the square root of the Average Variance Extracted (AVE).

**Table 4:** Discriminant validity (Heterotrait-Monotrait Ratio).

	CT	IP	TP	TW
CT				
IP	0,521			
TP	0,475	0,847		
TW	0,706	0,559	0,560	
UE	0,687	0,472	0,490	0,750

#### 5.3.2 Structural model

The results confirm the strong positive effect of the user experience in serious games on critical thinking and teamwork. This result joins the research that highlights the importance of serious games to learn soft skills. Concerning critical thinking, our results show its strong positive effect on individual performance. Teamwork has a significant positive effect on individual performance.

**Figure 2:**
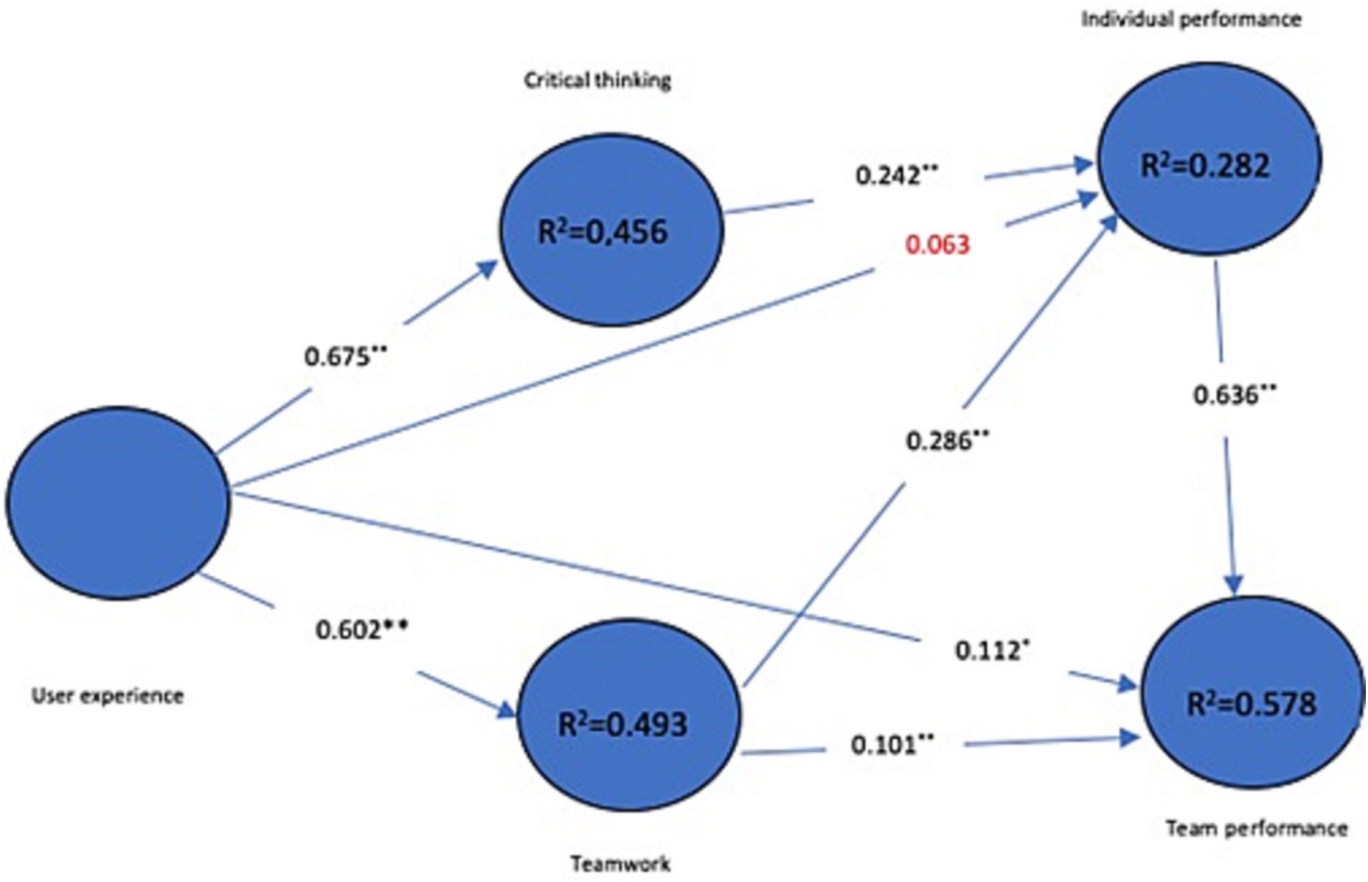
Structural model.

In terms of *R*^2^, results show that the user experience explains 45.6% of critical thinking. The user experience explains 49.3% of teamwork. User experience, critical thinking and teamwork explain 28.2% of the individual performance. Finally, individual performance, teamwork and the user experience explain 57.8% of the team performance. As shown in Figure 2, User Experience in serious games contributes significantly and positively to critical thinking (pc = 0.675), teamwork (pc = 0.602) and team performance (pc = 0.112). The user experience fosters the acquisition of new soft skills mainly when the game is played in a team. However, this user experience has no significant effect on individual performance (pc = 0.063). Critical thinking has a significant and positive influence on individual performance (pc = 0.242), which means that critical thinking could be a motivator for better in-dividual performance. Individual performance is influenced by teamwork (pc = 0.286). In-dividual performance has a positive impact on team performance (pc = 0.636).

Except for the association between team performance and user experience (<0.05), all *p*-values are less than 0.01. In addition, user experience and individual performance have no significant relationship.

#### 5.3.3 Data groups

Our main findings regarding the group before (the end of the game) are as follows:

- The correlation between user experience and team performance was not significant (pc = 0.071).
- The *R*^2^ of individual performance was very low 0.118 (<0.25).
- The correlation between teamwork and team performance was not significant (pc = 0.073).
- The correlation between individual performance and team performance was very significant (pc = 0.721).

Our main findings regarding the group after (the end of the game) are as follows:

- The correlation between user experience and team performance becomes significant (pc = 0.150).
- The R^2^ of individual performance rises 0.542 (>0.25).
- The correlation between teamwork and team performance becomes significant (pc = 0.265).
- The correlation between teamwork and individual performance rises from (pc = 0.125) before the end of the game to (pc = 0.373) after the end of the game.
- The correlation between critical thinking and individual performance rises from (pc = 0.182) before the end of the game to (pc = 0.425) after the end of the game.
- The correlation between individual performance and team performance decreases to (pc = 0.439).
- The correlation between user experience and critical thinking rises from (pc = 0.661) before the end of the game to (pc = 0.694) after the end of the game.
- The correlation between user experience and teamwork rises from (pc = 0.549) before the end of the game to (pc = 0.655) after the end of the game.

### 5.4 Qualitative analysis

After the game session ended, we invited students to give their opinion about the game experience and to report criticism, remarks, and feelings. The overall responses were positive, and students are happy with their experience. Several anonymous statements were expressing satisfaction and happiness. They are glad to experience new learning material and to learn useful skills such as decision making, team collaboration, etc. The experience is “great” “interesting” “instructive” and “exciting, One student wrote “It’s really appealing for me because it is my first experience of business simulation”!

Students appreciate that “The game encourages us to work together,” “the game is good for working in groups and develop business mind and relationship” “teamwork is very good, and we always made the decisions together” and an “opportunity to exchange our ideas, One student expressed that he likes the game so much “because it requires our abilities of logic and communication, Another found it interesting and “felt that I was more absorbed in that game than in a traditional class, Students report that “have learned a lot through this business game, They qualify the experience as learning by doing experience and argue that “it is a very good way to learn easily and faster, Another student found the game as “a very interesting way to enter the world of business’ ‘. Another wrote that the game gives him “a completely different attitude toward business” and that this experience fosters his attitude toward business. According to one student, “This game made me master new skills such as leading group work and dispatching tasks, Another student was surprised that the game experience seems to be very realistic with so many obstacles and parameters to be considered when starting a new business. According to them, the game is instructive and requires several skills, the most important things are communication and the discipline toward team goals and commitment. Some students are expressing their need for a beforehand knowledge to avoid time wasting in searching for some financial and accounting details. They require a seminar explaining some key concepts, pitfalls to avoid and a time schedule to work. For them such a class is becoming a must have, however they want the game to be scheduled for more than 4 days to work without pressure and stress. Students did not like the random dispatching of team members and were seeking to organize their team on their own.

In order to categorize the students’ opinions, we used a platform called MonkeyLearn, which analyzes these opinions using a machine learning method and provides us with a set of results as illustrated in Table 5. Indeed, this latter summarizes the most used keywords in the students’ responses.

**Table 5:** Students’ opinions categorization.

Keywords	Times
Good experience, great experience, nice experience	30 times
Learning, instructive	35 times
Funny, fun, cool	23 times
Like, enjoy, pleased	33 times
Interesting, thinkful	19 times
Team, group, collaboration	29 times
Studies, business world	12 times

Based on these results, three classes of students’ opinions emerged. The first class considers the serious games as a good learning and instructive experience that brings a completely different attitude toward business. The second one focused on the fun and joy dimension of using serious games. The last class found the serious games interesting and thoughtful, which allows working with teammates pretty well, and to be in competition alongside other teams was a great opportunity. Findings of the qualitative analysis sustain our hypothesis regarding beneficial effects of serious games on students’ soft skills including critical thinking and teamwork.

## 6 Discussion

With the rapid evolution of technology and the momentum of active teaching, using Serious Games in higher education is a part of new teaching methods to enhance graduates’ performance. Moreover, these advanced tools may also be fruitful to prepare skilled individuals for the professional environment. Serious games are real tools for knowledge, learning or progression. They are “valuable” video games put in service by professionals. They are emerging and established as real means of communication: a modern medium, flexible, and easy to access with the presence of the Internet and the explosion of interactive entertainment. Researchers encourage the use and the enrollment of serious games in university because of their assets as sophisticated simulations per-mitting a learning by doing experience. These games are proving their success in hard skills and start to expand to develop soft skills.

The current paper presents a quantitative and qualitative study that tries to focus on the impact of user experience in serious games on soft skills mainly on critical thinking and teamwork. The results reveal that the experience with games is more than interesting to develop soft skills. Indeed, positive, and significant influences are reported on all be-fore mentioned soft skills. Consequently, serious gaming is proving their success by supporting teamwork and critical thinking.

Regarding the nature of serious games used in the simulation, students are asked to make decisions about their startups, they need to conduct deep discussions, to perform calculations and learn from their own mistakes. The game raises the difficulty after each decision and endorses new problems and sometimes drives the students to commit errors. After each decision, students receive the results after the game processes all decisions. Here, they must take a step back to analyze their results regarding their decisions and learn new lessons. Critical thinking is driving the students to better analyze and communicate. Indeed, the better teams are those who communicate better and synergize their efforts toward the success of the next decision.

In addition, critical thinking is found to have a positive and significant effect on individual performance which is in coherence with the literature. Critical thinking is mediating the effect of user experience on individual performance. Since user experience has an insignificant direct effect on individual performance but it has an indirect effect passing through critical thinking. In fact, critical thinking is a “must have” skill needed by leaders and followers in the aim to better develop communication, innovation, and creativity in the team (Banutu-Gomez, 2004). Indeed, a critical thinker is an open-minded person who is careful about his beliefs and thoughts and then more punctual in the manner he behaves (Piel, 2008). Consequently, developing critical thinking inside teams reduces conflicts, drives deeper communication regarding decision making and allows a better team cohesion and effectiveness. It enables employees to work effectively with others, adapt to new challenges, and contribute positively to the organization’s culture (Haselberger et al., 2012; Gaurav et al., 2022). It enhances communication inside the team and fosters individual learning effectiveness (Boyd and Fales, 1983). The findings of this part are joining the literature in (Boyd and Fales, 1983; Hollenbeck et al., 1995; Natale and Ricci, 2006).

Better communication and synergy in a team creates successful team working and impacts positively and significantly individual and team performance. In fact, altruistic and helpful team members are more sociable and givers, which creates a very cohesive and cooperative team. The overall context has a significant impact on individuals and may influence their performance. Results confirm that teamwork positively influences individual performance. This latter is a tremendous parameter that may influence the success of teamwork and team performance. According to our results, critical thinking and teamwork are both having a direct effect on individual performance.

Furthermore, the user experience has an indirect effect on individual performance via critical thinking and teamwork. These two constructs constitute the means by which the game is enhancing the individual performance of team members. Hence, the impact of the game on individual performance is fostered by teamwork and critical thinking. Here we understand that serious games are helping the students to develop better team-working and enhance their critical thinking skills. These two constructs are crucial for the development of individuals and the success of teams and organizations.

In addition, User experience is having both direct effect and indirect effect on team performance through teamwork. It means that students are teamwork is a strong mediating construct of the relationship between user experience and team performance. Students are learning to collaborate better through the game, which enhances the whole performance of the team.

Intuitively, that learning experience within the game has the biggest impact on the outcomes (soft skills) however other items such as gaming experience, adaptivity and fidelity would influence the overall experience (roughly all path coefficients are superior to 0.7). For example, Adaptivity is the manner in which the game adapts itself with the needs and the goals of the learner. So, it has an added value to enhance the outcomes of the game.

Furthermore, regarding our data groups (before and after the end of the game) we can tell that this gaming experience enhances team performance as the game is advancing and the students understand that they must collaborate efficiently which rises the impact of user experience on team performance.

At the beginning of the game, the individual performance was weakly explained by critical thinking, user experience and teamwork which raised until the end of the game. In addition, the correlation between teamwork and team performance was not significant because students were learning step by step through professors’ guidance and several tips and hints to enhance the collaboration between teammates, mainly tasks’ repartition and time management. The correlation between individual performance and team performance was very significant and decreases as the game advances to make more place to teamwork. It means that individual performance is no longer impacting team performance. As the game becomes more difficult, synergy is crucial, students make more effort in teams than individually.

By the end of the game, critical thinking becomes more influential on individual performance as students understand the importance of this soft skill and learn through professors’ recommendations and hints how to make better decisions and to privilege a strategy with better profit in the long run and avoid making any decision without calculating several crucial key factors.

## 7 Conclusion

Nowadays, organizations are more interested in team performance and soft skills in addition to hard skills, serious games represent a new way to develop both skills and foster youth employability. According to the literature of MMOG games, gamers are developing new skills such as leadership, community management, communication, teamworking, etc. and then Wasko et al. (2011) wrote “Today, most human resource managers would be surprised if a job applicant’s CV were to come across his or her desk in which the applicant lists “WoW Guild Leader” as a work-related experience.”

Finally, in addition to hard skills (accounting, operation management, marketing, inventory management) and soft skills (teamwork and critical thinking), students were more than enchanted with the experience. At the end of the session, we invited them to present their opinions about the game. They find the game familiar and less stressful compared to traditional courses. They feel satisfied and happy about this “innovative experience” and state that this kind of game is becoming a “must have, They find the experience collaborative and exciting thanks to the rising level of difficulty proposed by the game. Students are enjoying synergizing their efforts to compete with other teams. They highlighted the importance of communication and teamwork to succeed in this game. Students are feeling engaged and “appropriating” the game as if they are having their startup for real. They are enrolled in the scenario to the extent to which we saw emotions and tears when presenting the results to the winners.

The respondents are registered in the same curriculum, and our sample is homogeneous. They have the same experience with games besides they belong to two different colleges installed in KSA and France with different cultures and environments.

A primary research limitation is associated with the homogeneous sample. Students are the same age. Even if the sample is divided into students from KSA and France, the same results are reported for both. This business game is only a simulation that tries to approach the real world but it’s not the reality of startups.

Students are the main target audience of this kind of game. Studying their attitude, behavior, reactions may be a valuable added value to teachers. In this paper we try to understand their behavior regarding the use of serious games as an educational tool to deliver knowledge and develop soft skills. Students need to be empowered to be a stakeholder in the choice of their learning strategies and materials. They could bring their own judgment and new perceptions that cannot be seen or perceived by teachers or pedagogues. In the next step, we need to expand our research on more soft skills such as communication, cognitive engagement, and team performance. We are required to consider heterogeneous samples with different ages and experiences. We need to use the game with professionals to bring deeper insights into the real impact of these games on soft skills development.

## Data availability statement

The original contributions presented in the study are included in the article/supplementary material, further inquiries can be directed to the corresponding author.

## Ethics statement

The studies involving humans were approved by Institutional Review Board (IRB) Princess Nourah bint Abdulrahman University, Riyadh, KSA. The studies were conducted in accordance with the local legislation and institutional requirements. At the beginning of the survey, the participants were informed that no personal information is included, and by filling it they give their informed consent to participate in this work.

## Author contributions

NY: Conceptualization, Data curation, Formal analysis, Methodology, Resources, Supervision, Supervision, Writing – review & editing. IB: Conceptualization, Data curation, Formal analysis, Investigation, Methodology, Resources, Software, Supervision, Validation, Visualization, Writing – original draft, Writing – review & editing. LJ: Conceptualization, Data curation, Formal analysis, Funding acquisition, Investigation, Methodology, Project administration, Resources, Software, Supervision, Visualization, Writing – original draft, Writing – review & editing. NA: Conceptualization, Software, Validation, Visualization, Writing – review & editing.
